# Rapid subtype-specific detection of respiratory syncytial virus A and B using an RNase HII-dependent RT-LAMP lateral flow assay

**DOI:** 10.3389/fchem.2026.1942908

**Published:** 2026-09-10

**Authors:** Woong Sik Jang, Eunji Lee, Sun-Young Ko, Chae Seung Lim

**Affiliations:** 1 Department of Laboratory Medicine, College of Medicine, Korea University Guro Hospital, Seoul, Republic of Korea; 2 Department of Emergency Medicine, College of Medicine, Korea University Guro Hospital, Seoul, Republic of Korea; 3 BK21 Graduate Program, Department of Biomedical Sciences, College of Medicine, Korea University, Seoul, Republic of Korea; 4 Department of Laboratory Medicine, Korea University Ansan Hospital, Korea University College of Medicine, Seoul, Republic of Korea

**Keywords:** blocked inner primer, lateral flow assay, point-of-care diagnostics, respiratory syncytial virus subtyping, RhPCR, RT-LAMP

## Abstract

**Introduction:**

Rapid and simultaneous detection of respiratory syncytial virus A (RSV A) and RSV B with subtype discrimination is important for point-of-care (POC) diagnostics and epidemiological surveillance.

**Methods:**

We developed the RSV A/B rh-RT-LAMP-NALF assay, a duplex reverse transcription loop-mediated isothermal amplification (RT-LAMP) system combined with nucleic acid lateral flow (NALF) detection for simultaneous subtype-specific detection of RSV A and RSV B from nasopharyngeal swab specimens. To improve specificity during multiplex RT-LAMP, blocked inner primers, activated by RNase HII, were incorporated to suppress non-specific amplification. Subtype-specific detection was achieved on a single PCRD multiplex strip using 5′-FAM–and 5′-DIG–labeled loop primers for RSV A (T1 line) and RSV B (T2 line), respectively.

**Results:**

The analytical limit of detection was 4 × 10^1^ copies/μL using plasmid DNA standards. In clinical specimen dilution series, the assay detected RSV down to the 10^−3^ dilution in clinical specimens, showing sensitivity approaching that of the comparator RT-PCR assay. Clinical validation was performed using 281 archived nasopharyngeal swab specimens (87 RSV A-positive, 94 RSV B-positive, and 100 RSV-negative), with the Allplex™ Respiratory Panel 1 assay used as the reference method. The RSV A/B rh-RT-LAMP-NALF assay demonstrated sensitivities of 95.4% (95% CI: 88.8%–98.2%) for RSV A and 97.9% (95% CI: 92.6%–99.4%) for RSV B, with no false-positive results observed in the 100 RSV-negative specimens. These results were comparable to those of the Allplex™ SARS-CoV-2/FluA/FluB/RSV assay (RSV A: 95.4%; RSV B: 98.9%) and higher than those of the STANDARD Q RSV Ag Test (RSV A: 82.8%; RSV B: 92.6%). Limited cross-subtype reactivity was observed in one RSV A-positive specimen and one RSV B-positive specimen, both of which showed dual T1/T2 signals on the lateral flow strip. No cross-reactive signals were observed in 42 specimens representing 14 common respiratory pathogens other than RSV.

**Discussion:**

Overall, the RSV A/B rh-RT-LAMP-NALF assay provides a rapid, accurate, and subtype-specific POC diagnostic platform for RSV detection and surveillance.

## 1 Introduction

Respiratory syncytial virus (RSV) is a major cause of acute lower respiratory tract infection worldwide and is responsible for substantial morbidity and mortality in both pediatric and adult populations. Globally, RSV is estimated to cause approximately 33 million lower respiratory tract infection episodes, 3.2 million hospitalizations, and more than 100,000 deaths annually among children under 5 years of age (Shi et al., 2017). In addition, RSV infection represents a significant clinical burden in elderly and immunocompromised individuals, with approximately 14,000 RSV-associated deaths reported annually among adults aged 65 years or older in the United States alone (Falsey et al., 2005). RSV is classified into two major subtypes, RSV A and RSV B, which co-circulate during seasonal outbreaks with varying prevalence depending on geographic region and epidemic season. These subtypes may differ in transmissibility, clinical severity, and susceptibility to emerging vaccines and antiviral strategies (Agoti et al., 2017; Nuttens et al., 2024). Consequently, subtype-specific identification of RSV has become increasingly important not only for epidemiological surveillance and outbreak monitoring but also for evaluating the impact of recently introduced RSV vaccines and monoclonal antibody prophylactic agents on subtype distribution.

Rapid antigen detection tests (RDTs) are widely used in point-of-care settings because they provide rapid results without requiring specialized instrumentation. However, the pooled sensitivity of RSV antigen tests is reported to be approximately 80% overall and decreases substantially in specimens with low viral loads and in adult patient populations (Chartrand et al., 2015). Furthermore, most commercially available RSV RDTs do not distinguish between RSV A and RSV B, thereby limiting their utility for subtype surveillance. Reverse transcription quantitative PCR (RT-qPCR) remains the reference standard for RSV diagnosis because of its superior analytical sensitivity and subtype discrimination capability. Nevertheless, its reliance on thermal cycling instruments, centralized laboratory infrastructure, and trained personnel restricts its implementation in decentralized or resource-limited settings.

Loop-mediated isothermal amplification (LAMP), first described by Notomi et al. (2000), has emerged as a promising molecular diagnostic platform for point-of-care testing (POCT) because of its rapid amplification kinetics, isothermal reaction conditions, and minimal instrumentation requirements. Integration of reverse transcription LAMP (RT-LAMP) with nucleic acid lateral flow (NALF) detection further enhances its suitability for decentralized diagnostics by enabling visually interpretable results without optical detection equipment. Lateral flow–based detection systems have been widely adopted as low-cost and instrument-free diagnostic platforms, and considerable efforts have been directed toward improving their analytical sensitivity and multiplexing capability (Lamprou et al., 2025; Zheng et al., 2021). However, multiplex RT-LAMP assays remain technically challenging because simultaneous amplification using multiple primer sets substantially increases the risk of non-specific amplification and inter-primer interactions, which can generate false-positive or cross-reactive signals on lateral flow strips. These non-specific amplification products represent one of the major limitations restricting the practical application of multiplex RT-LAMP-NALF systems.

To address this limitation, we applied the principle of RNase HII-dependent PCR (rhPCR), originally developed by Dobosy et al. (2011), to the RT-LAMP primer system. In rhPCR, primers containing a single ribonucleotide residue and a 3′ blocking group are activated only after RNase HII-mediated cleavage upon correct target hybridization, thereby suppressing non-specific amplification. Although rhPCR has previously been applied to multiplex PCR and SNP discrimination assays, and probe-based detection strategies have been incorporated into real-time multiplex LAMP systems (Tanner et al., 2012), to our knowledge, the application of rhPCR to improve specificity in a multiplex RT-LAMP lateral flow assay has not been previously reported.

In this study, we developed an RSV A/B rh-RT-LAMP-NALF assay for simultaneous subtype-specific detection of RSV A and RSV B from nasopharyngeal swab specimens. To improve specificity in multiplex RT-LAMP-NALF, rhPCR-based blocked inner primers were incorporated into the assay to enable RNase HII-dependent target-specific primer activation and suppression of non-specific amplification. Subtype-specific detection was achieved on a single lateral flow strip using 5′-FAM– and 5′-DIG–labeled loop primers for RSV A (T1 line) and RSV B (T2 line), respectively. Analytical sensitivity, subtype specificity, cross-reactivity, and clinical diagnostic performance were evaluated using 281 archived clinical specimens, with a commercial antigen-based RDT and a commercial multiplex RT-PCR assay included as comparator methods.

## 2 Materials and methods

### 2.1 Clinical samples and nucleic acid extraction

A retrospective panel of 281 archived nasopharyngeal (NP) swab specimens was obtained from Korea University Guro Hospital (Seoul, Republic of Korea). The specimens had originally been collected between January 2018 and July 2019 during routine clinical care and consisted of 87 RSV A–positive, 94 RSV B–positive, and 100 RSV-negative samples. RSV positivity, negativity, and subtype classification were based on the results of the Allplex™ Respiratory Panel 1 assay (Seegene, Seoul, Republic of Korea), which had been performed as part of routine clinical diagnosis at Korea University Guro Hospital before the present study. The specimens were not consecutively included but were retrospectively selected to constitute predefined RSV A-positive, RSV B-positive, and RSV-negative groups. Specimens were eligible for inclusion when the routine Allplex™ Respiratory Panel 1 result was available, the corresponding residual NP specimen was available, and the residual specimen volume was sufficient to perform nucleic acid extraction and all study assays. Specimens with incomplete diagnostic information, insufficient residual volume, or inadequate specimen quality were not included. After the study panel had been established, no specimens were excluded because of inadequate quality, insufficient volume, or assay failure. These diagnostic classifications were established independently of the RSV A/B rh-RT-LAMP-NALF assay evaluated in the present study. To comparatively evaluate the diagnostic performance of the RSV A/B rh-RT-LAMP-NALF assay, two additional comparator assays were performed using the same specimen set. The STANDARD Q RSV Ag Test (SD Biosensor, Gyeonggi-do, Republic of Korea), a rapid antigen detection test (RDT), was included as an antigen-based comparator assay. In addition, the Allplex™ SARS-CoV-2/FluA/FluB/RSV assay (Seegene, Seoul, Republic of Korea), a multiplex molecular diagnostic assay capable of RSV detection without subtype differentiation, was included as a molecular comparator assay for direct performance comparison. The RSV-negative group consisted exclusively of specimens in which no respiratory pathogen was detected and was included to evaluate assay specificity and false-positive reactivity. Total nucleic acids were extracted from all NP swab specimens using the AdvanSure™ E3 System (Invitros, Cheongju-si, Republic of Korea) according to the manufacturer’s instructions. Briefly, 200 µL of each clinical specimen was loaded into a 96-well extraction plate and processed using the automated extraction protocol. Extracted nucleic acids were stored at −80 °C until use. This retrospective study utilized residual clinical specimens obtained during routine diagnostic testing. The study was conducted in accordance with the Declaration of Helsinki and approved by the Institutional Review Board of Korea University Guro Hospital (approval number: 2019GR0055), which granted both a waiver of informed consent and exemption from additional ethical review.

### 2.2 NALF-LAMP primer design and labeling

**FIGURE 1:**
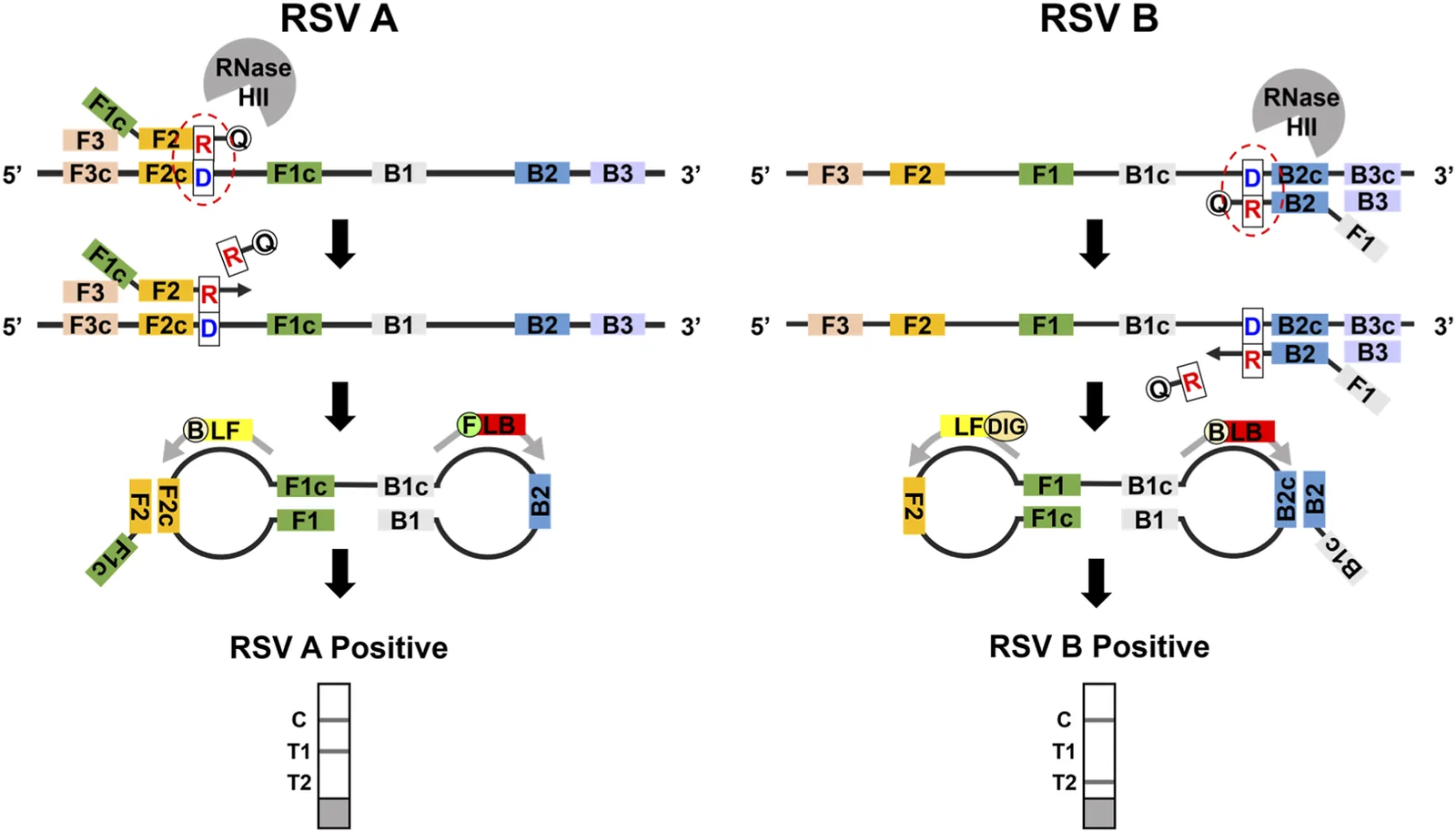
Schematic illustration of the RSV A/B rh-RT-LAMP-NALF assay principle (Left) RSV A detection; (Right) RSV B detection. Blocked inner primers (FIP for RSV A; BIP for RSV B**)** containing a single ribonucleotide residue and a 3′-BHQ1 blocking group are activated by RNase HII cleavage upon target hybridization, enabling target-dependent LAMP amplification. The resulting amplicons incorporate 5′-FAM (RSV A) or 5′-DIG (RSV B) loop primers and are detected on a PCRD multiplex lateral flow strip at the T1 (anti-FAM) or T2 (anti-DIG) line, respectively. BHQ1, Black Hole Quencher 1; DIG, digoxigenin; FAM, 6-carboxyfluorescein; C, control line; T1, RSV A detection line; T2, RSV B detection line.

The target-binding sequences of the RSV A LAMP primers, directed against the matrix (M) gene, were derived from a previously published multiplex LAMP assay (Mahony et al., 2013). The target-binding sequences of the RSV B LAMP primers, directed against the RNA-dependent RNA polymerase (L) gene, were previously developed by our group for a real-time fluorescence RT-LAMP assay and reported by Lim et al. (2026). For the present assay, the RSV A FIP and RSV B BIP were converted into RNase HII-dependent blocked primers by adding rA–CTTA–BHQ1 and rA–TTTG–BHQ1 to their 3′ends, respectively. Reporter-labeled loop primers were also incorporated to enable duplex amplification and subtype-specific lateral flow detection (Table 1). Potential secondary structures and primer-dimer formation within the complete duplex primer set were evaluated using Primer3-py v2.3.0 (Supplementary Table S1). RSV A and RSV B amplicons were differentiated on a single PCRD multiplex lateral flow strip (Abingdon Health, York, UK) through orthogonal hapten capture using an anti-FAM antibody-coated T1 line for RSV A detection and an anti-DIG antibody-coated T2 line for RSV B detection. A streptavidin-coated concentration zone positioned upstream of the test lines captured biotin-labeled amplicons prior to T1/T2 detection, while a biotin–anti-biotin control line (C line) was used to verify appropriate sample migration and strip integrity. To suppress non-specific amplification arising from the high primer concentration inherent to duplex LAMP, rhPCR-based blocked inner primers were incorporated following the principle described by Dobosy et al. (2011). A single ribonucleotide residue (rA) and a 3′-BHQ1 blocking group were introduced into the FIP of the RSV A primer set and the BIP of the RSV B primer set. In the absence of complementary target hybridization, the 3′-BHQ1 block prevented extension by Bst DNA polymerase. Upon sequence-specific hybridization, RNase HII cleaved the RNA–DNA hybrid at the ribonucleotide residue, thereby removing the 3′block and enabling target-dependent primer extension. For RSV A detection, the loop-backward (LB) primer was labeled with 5′-FAM and the loop-forward (LF) primer with 5′-biotin. For RSV B detection, the LF primer was labeled with 5′-DIG and the LB primer with 5′-biotin. During loop-mediated extension, the labeled loop primers were incorporated into the amplification products, generating dual-labeled amplicons containing both hapten and biotin tags. Following amplification, streptavidin-gold conjugates bound to the biotin-labeled amplicons, and the resulting complexes were captured at the T1 or T2 line according to the incorporated hapten label, generating subtype-specific detection signals. The principles of RNase HII-dependent blocked primer activation and subtype-specific detection on the PCRD multiplex lateral flow strip are schematically illustrated in Figure 1.

**TABLE 1:** Primer sequences and modifications used in the duplex RSV A/B rh-RT-LAMP-NALF assay.

Primer set	µM	Sequence (5'→3′)	Modification
RSV A primers mix (20× mix)			
F3	4	GCT​GTT​CAA​TAC​AAT​GTC​CTA​GA	–
B3	4	GGT​AAA​TTT​GCT​GGG​CAT​T	–
FIP (rh-LAMP)	32	TCTGCTGGCATGGATGATTGG-AGACGATGATCCTGCATCaCTTA-BHQ1	3′-BHQ1 (rh probe)
BIP	32	CTA​GTG​AAA​CAA​ATA​TCC​ACA​CCC​A-GCA​CTG​CAC​TTC​TTG​AGT​T	–
LF	10	Biotin-ACATGGGCACCCATATTGTAAG	5′-Biotin
LB	10	FAM-AGGGACCTTCATTAAGAGTCATGAT	5′-FAM
RSV B primers mix (20× mix)			
F3	4	TTTGGTGGTGGTGATCCT	–
B3	4	CAA​ACT​CGG​CAT​TTG​GAT​T	–
FIP	32	AGC​TCA​ACA​CAA​ACA​CTG​AAT​GTA​C-TAT​ATC​GAA​GCT​TTT​ATA​GGA​GAA​C	–
BIP (rh-LAMP)	32	CTGGTCACGATCTACAAGATAAGC-TGTGATGACACAAGTCAAGAaTTTG-BHQ1	3′-BHQ1 (rh probe)
LF	10	DIG-TATAGCTTCTGTAAGGAAGTCTGGA	5′-DIG
LB	10	Biotin-GGATCTTCCAGATGATAGACTGAAC	5′-Biotin

^a^FIP (RSV A) and BIP (RSV B) carry an internal ribonucleotide residue (lowercase ‘a’) and a 3′-BHQ1 blocking group, constituting rhPCR-based blocked inner primers activated by RNase HII upon target hybridization. BHQ1, Black Hole Quencher 1; DIG, digoxigenin; FAM, 6-carboxyfluorescein; rhPCR, RNase HII-dependent PCR.

### 2.3 RSV A/B NALF rh-RT-LAMP assay

Each RT-LAMP reaction was prepared in a total volume of 25 μL, containing 12.5 µL of 2× RT-LAMP reaction mix (ELPIS Biotech, Daejeon, Korea) incorporating Bst DNA polymerase and MMLV reverse transcriptase, 1.25 µL each of RSV A and RSV B primer mixes (20× working concentration), 0.5 µL of RNase HII (Integrated DNA Technologies, Coralville, IA, United States; 5 U/μL final concentration), 2.0 µL of template RNA, and nuclease-free water to 25 µL. RNase HII enables target-dependent cleavage of the ribonucleotide residue in the blocked FIP (RSV A) and BIP (RSV B) inner primers. Reaction assembly was performed on ice in a dedicated pre-amplification area using aerosol-resistant pipette tips. Template nucleic acid, extracted and stored as described in Section 2.1, was used directly; 2 µL of eluate was added as template per reaction. Amplification was performed at 62 °C for 30 min in a CFX96 Touch real-time PCR system (Bio-Rad Laboratories, Hercules, CA, United States), with fluorescence data acquired at 1-min intervals to monitor reaction kinetics. All reactions included a no-template control (NTC) assembled with nuclease-free water substituted for template RNA. Following completion of the 30-minute RT-LAMP reaction, 10 µL of amplification product was diluted into 140 µL of PCRD running buffer (Abingdon Health) and applied immediately to the sample port of the PCRD multiplex lateral flow strip. The strip consists of the T1 test line (anti-FAM antibody–colloidal gold conjugate) for RSV A detection, the T2 test line (anti-DIG antibody–colloidal gold conjugate) for RSV B detection, and a control (C) line for confirmation of strip function. Results were read by unaided visual inspection at exactly 5 min after sample application. Each line was scored independently as: positive (+), clearly visible colored band; weak positive (W+), faint but unambiguously discernible band; or negative (−), no visible band. W+ results were classified as positive in all statistical analyses. A run was considered invalid and repeated if the C line was absent.

### 2.4 Comparative RT-PCR assay

All specimens were additionally tested using the Allplex™ SARS-CoV-2/FluA/FluB/RSV Assay (Seegene Inc., Seoul, Republic of Korea) as a comparative molecular assay. This assay simultaneously detects influenza A, influenza B, SARS-CoV-2, and RSV in a single reaction; however, RSV is detected as a single target without subtype differentiation. This assay was selected to enable comparison of the overall RSV detection performance of the RSV A/B rh-RT-LAMP-NALF assay against a validated molecular diagnostic platform. RT-PCR was performed according to the manufacturer’s instructions on a CFX96 Touch real-time PCR system. RSV positivity and negativity were automatically determined by the manufacturer’s proprietary analysis software (Seegene Viewer), and the cycle threshold (Ct) value was recorded for each RSV-positive specimen. All RSV A/B rh-RT-LAMP-NALF assay results were interpreted without knowledge of the comparative RT-PCR results.

### 2.5 Comparative RDT assay

All specimens were independently tested using the STANDARD Q RSV Ag Test (SD Biosensor, Gyeonggi-do, Republic of Korea), a commercially available rapid antigen detection test (RDT), as a comparator. This RDT detects RSV nucleoprotein antigen and reports a binary RSV-positive or RSV-negative result without subtype discrimination. Testing was performed according to the manufacturer’s instructions using the same archived NP swab eluates as those used for the RSV A/B rh-RT-LAMP-NALF assay. RDT operators were blinded to all NALF and reference RT-PCR results at the time of testing, and RDT results were recorded before any unblinding occurred.

### 2.6 Analytical sensitivity assessment

Analytical sensitivity was assessed using two complementary reference materials: synthetic plasmid DNA standards and serially diluted clinical specimens. Plasmid DNA constructs containing the LAMP target regions for RSV A and RSV B were obtained commercially and quantified using a NanoDrop spectrophotometer (Thermo Fisher Scientific). The copy number concentration was calculated from the measured DNA concentration using the following formula: Copies/μL = (DNA concentration (ng/µL) × 6.022 × 10^23^)/(plasmid size (bp) × 660 × 10^9^), where 6.022 × 10^23^ is Avogadro’s number, 660 g/mol is the average molecular weight per base pair, and 10^9^ is the conversion factor from nanograms to grams. Serial 10-fold dilutions were prepared in nuclease-free water from 4 × 10^5^to 4 × 10^−2^ copies/μL, and each dilution was tested in three independent NALF runs. To assess analytical sensitivity under clinically relevant conditions accounting for matrix effects and RNA template complexity, high-titer clinical specimens positive for RSV A or RSV B by the Allplex™ Respiratory Panel 1 reference assay (Ct ≤ 22) were serially diluted 10-fold in RSV-negative NPS matrix from 10^−1^ to 10^−7^, and each dilution was similarly tested in triplicate. The corresponding Allplex™ Respiratory Panel 1 Ct value at each dilution was determined in parallel to establish the relationship between dilution factor and viral load. The analytical sensitivity was defined as the lowest concentration or dilution at which all 3 of 3 replicate NALF runs yielded a positive result (T1 or T2 band scored as W+ or higher). Cross-subtype signal (T2 positivity in RSV A standards; T1 positivity in RSV B standards) was also monitored across all dilutions to confirm analytical specificity.

### 2.7 Statistical analysis

Diagnostic sensitivity and specificity with 95% confidence intervals (CI) were calculated from standard 2 × 2 contingency tables using the Allplex™ Respiratory Panel 1 assay (Seegene, Seoul, Republic of Korea) as the reference standard. Ninety-five percent CIs were computed using the Wilson score interval method. For analytical sensitivity assessment, Ct values from triplicate runs are expressed as mean ± standard deviation (SD). All statistical analyses were performed using Microsoft Excel (Microsoft Corporation, Redmond, WA, United States).

## 3 Results

### 3.1 Optimization of the RSV A/B rh-RT-LAMP-NALF assay

**FIGURE 2:**
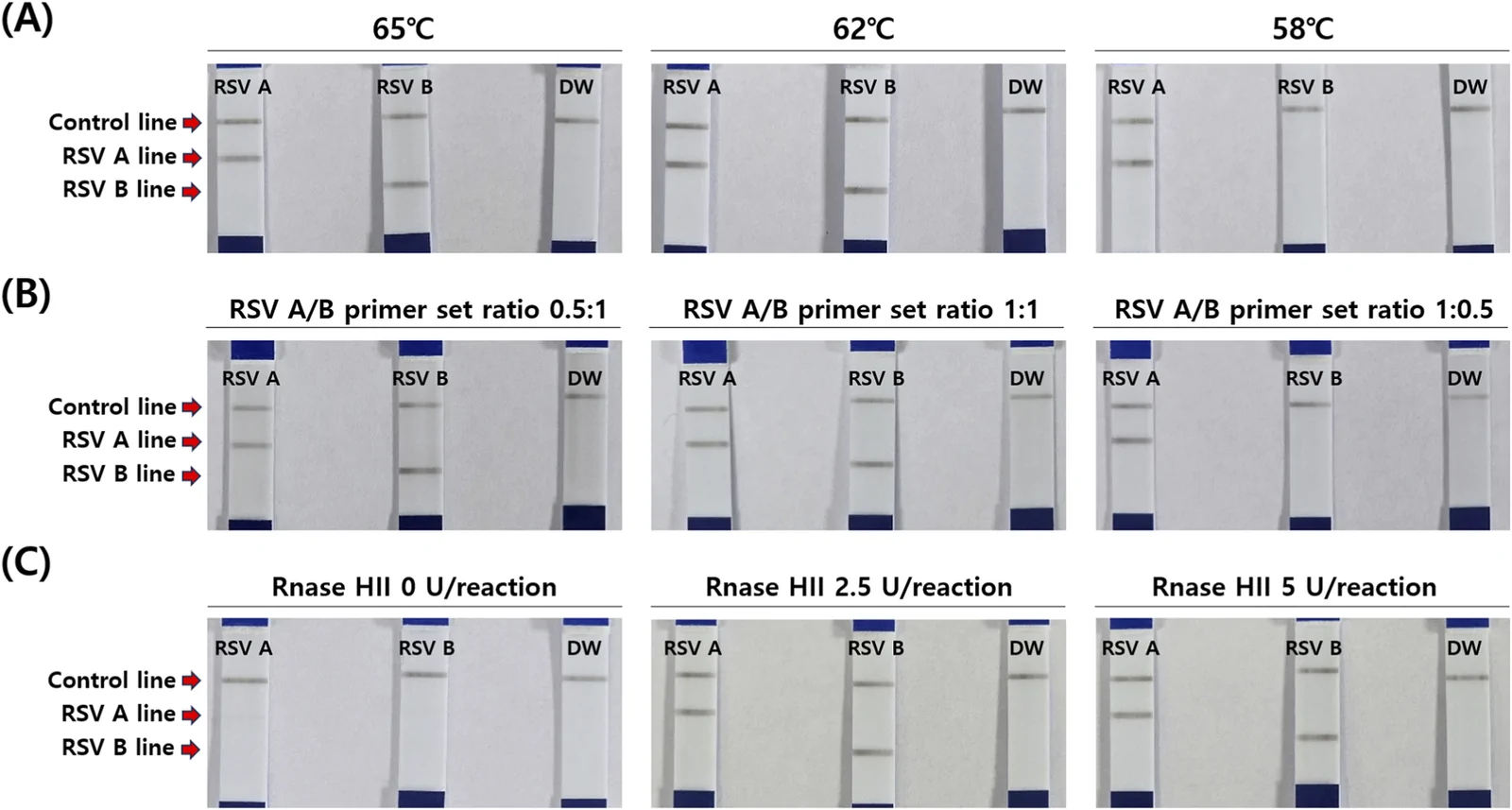
Optimization of reaction conditions for the RSV A/B rh-RT-LAMP-NALF assay. Lateral flow strip results are shown for RSV A template (left), RSV B template (center), and distilled water negative control (DW, right) under each condition. **(A)** Effect of reaction temperature (65 °C, 62 °C, and 58 °C). **(B)** Effect of RSV A:RSV B primer mix ratio (0.5:1, 1:1, and 1:0.5). **(C)** Effect of RNase HII concentration (0, 2.5, and 5 U/reaction). Absence of T1 and T2 signals at 0 U/reaction confirms that productive amplification is strictly dependent on RNase HII-mediated cleavage of the 3′-blocked inner primers. C, control line; T1, RSV A detection line; T2, RSV B detection line; DW, distilled water.

Optimization of the RSV A/B rh-RT-LAMP-NALF assay was performed using RSV A and RSV B plasmid templates, evaluating reaction temperature, primer mix ratio, and RNase HII concentration as key parameters (Figure 2). First, three reaction temperatures (65 °C, 62 °C, and 58 °C) were evaluated using a 1:1 primer mix ratio (Figure 2A). At 65 °C, signals were detected on both the T1 (RSV A) and T2 (RSV B) lines; however, band intensities were slightly lower than those observed at 62 °C. At 62 °C, both lines produced the strongest and most stable signals. At 58 °C, signal intensity was substantially reduced, particularly for the T2 (RSV B) line. Based on these results, 62 °C was selected as the optimal reaction temperature. Next, three RSV A:RSV B primer mix ratios (0.5:1, 1:1, and 1:0.5) were compared at 62 °C (Figure 2B). At a ratio of 0.5:1, the signal intensity of the T1 line (RSV A) decreased, whereas at a ratio of 1:0.5, reduced signal intensity was observed on the T2 line (RSV B). In contrast, the 1:1 ratio produced the strongest and most balanced band signals on both the T1 and T2 lines. Therefore, a primer mix ratio of 1:1 (RSV A:RSV B) was selected for subsequent experiments. Finally, RNase HII concentrations of 0, 2.5, and 5 U per reaction were evaluated (Figure 2C). In the absence of RNase HII, no signal was observed on either the T1 or T2 line, confirming that activation of the blocked inner primers and subsequent amplification were dependent on RNase HII activity. Comparable signal intensities were observed with both 2.5 U and 5 U of RNase HII. Therefore, 2.5 U was selected as the optimal concentration to minimize enzyme consumption while maintaining assay performance. Based on these findings, the optimal conditions for the RSV A/B rh-RT-LAMP-NALF assay were established as a reaction temperature of 62 °C, a primer mix ratio of 1:1 (RSV A:RSV B), and 2.5 U of RNase HII per reaction.

### 3.2 Analytical sensitivity

Analytical sensitivity was assessed using plasmid DNA standards and serially diluted clinical specimens (Table 2; Table 3). For plasmid DNA standards, the LOD of the RSV A/B rh-RT-LAMP-NALF assay was 4 × 10^1^ copies/μL for both RSV A (T1 line) and RSV B (T2 line). No cross-subtype signal was observed at any concentration tested for either RSV A or RSV B plasmid standards (Table 2). For clinically derived specimens, high-titer clinical RSV A and RSV B samples were serially diluted in RSV-negative NPS matrix and tested in triplicate alongside the Allplex™ SARS-CoV-2/FluA/FluB/RSV assay as a molecular comparator (Table 3). The clinical LOD of the RSV A/B rh-RT-LAMP-NALF assay was established at the 10^−3^ dilution for both RSV A and RSV B, corresponding to Allplex™ Ct values of 29.40 ± 0.28 and 28.26 ± 0.21, respectively. At the 10^−4^ dilution, the Allplex™ SARS-CoV-2/FluA/FluB/RSV assay continued to yield positive results for both RSV A (Ct 33.43 ± 0.28) and RSV B (Ct 32.37 ± 0.48), whereas the RSV A/B rh-RT-LAMP-NALF assay showed no detectable signal at the 10^−4^ dilution or lower, indicating that the analytical sensitivity of the RSV A/B rh-RT-LAMP-NALF assay is approximately one dilution step lower than that of the Allplex™ SARS-CoV-2/FluA/FluB/RSV assay. No cross-subtype signals were observed at any dilution in either specimen series.

**TABLE 2:** Analytical sensitivity of the RSV A/B rh-RT-LAMP-NALF assay using plasmid DNA standards.

Copies/μL	RSV A plasmid		RSV B plasmid	
	RSV A/B rh-RT-LAMP-NALF assay		RSV A/B rh-RT-LAMP-NALF assay	
	RSV A (T1 line)	RSV B (T2 line)	RSV A (T1 line)	RSV B (T2 line)
4 × 10^5^	Detected	N/D	N/D	Detected
4 × 10^4^	Detected	N/D	N/D	Detected
4 × 10^3^	Detected	N/D	N/D	Detected
4 × 10^2^	Detected	N/D	N/D	Detected
4 × 10^1^	Detected	N/D	N/D	Detected
4 × 10^0^	N/D	N/D	N/D	N/D
4 × 10^−1^	N/D	N/D	N/D	N/D
4 × 10^−2^	N/D	N/D	N/D	N/D
DW	N/D	N/D	N/D	N/D

N/D, not detected; DW, distilled water (no-template control). Detection was performed by visual interpretation of the PCRD lateral flow strip at 5 min post-amplification. All three independent replicates showed concordant results.

**TABLE 3:** Comparison of analytical sensitivity between the RSV A/B rh-RT-LAMP-NALF assay and the Allplex™ SARS-CoV-2/FluA/FluB/RSV assay using serially diluted clinical samples.

Dilution	RSV A clinical sample			RSV B clinical sample		
	Seegene assay (Ct ± SD)	RSV A/B rh-RT-LAMP-NALF assay		Seegene assay (Ct ± SD)	RSV A/B rh-RT-LAMP-NALF assay	
		RSV A (T1)	RSV B (T2)		RSV A (T1)	RSV B (T2)
1 × 10^−1^	21.90 ± 0.04	Detected	N/D	21.18 ± 0.15	N/D	Detected
1 × 10^−2^	25.30 ± 0.44	Detected	N/D	24.10 ± 0.16	N/D	Detected
1 × 10^−3^	29.40 ± 0.28	Detected	N/D	28.26 ± 0.21	N/D	Detected
1 × 10^−4^	33.43 ± 0.28	N/D	N/D	32.37 ± 0.48	N/D	N/D
1 × 10^−5^	N/D	N/D	N/D	N/D	N/D	N/D

Ct, cycle threshold; SD, standard deviation; N/D, not detected. Ct values represent the mean ± SD of three independent replicates. NALF results shown are the consensus of three replicates (Detected = 3/3 positive; N/D = fewer than 3/3 positive).

### 3.3 Clinical performance of the RSV A/B rh-RT-LAMP-NALF assay

The clinical performance of the RSV A/B rh-RT-LAMP-NALF assay was evaluated in comparison with the STANDARD Q RSV Ag Test (SD Biosensor, Gyeonggi-do, Republic of Korea) and the Allplex™ SARS-CoV-2/FluA/FluB/RSV assay (Seegene, Seoul, Republic of Korea) using 281 archived nasopharyngeal swab specimens, including 87 RSV A-positive, 94 RSV B-positive, and 100 RSV-negative samples, as defined by the Allplex™ Respiratory Panel 1 reference standard (Table 4). In the RSV A-positive group (n = 87), the RSV A/B rh-RT-LAMP-NALF assay demonstrated a sensitivity of 95.4% (83/87; 95% CI: 88.8%–98.2%) on the RSV A-specific T1 line, which was comparable to that of the Allplex™ SARS-CoV-2/FluA/FluB/RSV assay (95.4%; 83/87; 95% CI: 88.8%–98.2%) and higher than that of the STANDARD Q RSV Ag Test (82.8%; 72/87; 95% CI: 73.5%–89.3%). Cross-subtype reactivity on the RSV B-specific T2 line was observed in 0 of 87 RSV A-positive specimens, corresponding to a cross-subtype specificity of 100% (87/87; 95% CI, 95.8%–100%). In the RSV B-positive group (n = 94), the RSV A/B rh-RT-LAMP-NALF assay demonstrated a sensitivity of 97.9% (92/94; 95% CI: 92.6%–99.4%) on the RSV B-specific T2 line, which was comparable to that of the Allplex™ SARS-CoV-2/FluA/FluB/RSV assay (98.9%; 93/94; 95% CI: 94.2%–99.8%) and higher than that of the STANDARD Q RSV Ag Test (92.6%; 87/94; 95% CI: 85.3%–96.4%). Cross-subtype reactivity on the RSV A-specific T1 line was observed in 1 of 94 specimens, in which both T1 and T2 signals were detected on the lateral flow strip. However, confirmatory PCR amplification was observed only with the RSV B primer set, indicating that the T1 signal represented non-specific lateral flow reactivity rather than true RSV A amplification, corresponding to a cross-subtype specificity of 98.9% (95% CI: 94.2%–99.8%). In the RSV-negative group (n = 100), neither the RSV A/B rh-RT-LAMP-NALF assay nor the Allplex™ SARS-CoV-2/FluA/FluB/RSV assay produced false-positive results on either the T1 or T2 line, with both molecular assays achieving a specificity of 100% (95% CI: 96.3%–100%). In contrast, the STANDARD Q RSV Ag Test produced four false-positive results (specificity: 96.0%; 95% CI: 90.2%–98.4%), indicating lower specificity than the molecular assays. The clinical performance of all three assays is summarized in Table 4.

**TABLE 4:** Clinical performance of the RSV A/B rh-RT-LAMP-NALF assay, STANDARD Q RSV Ag Test (RDT), and Allplex™ SARS-CoV-2/FluA/FluB/RSV assay.

Clinical samples	​	Allplex™ SARS- CoV-2/FluA/FluB/RSV assay	STANDARD Q RSV Ag Test	RSV A/B rh-RT-LAMP-NALF assay	
				RSV A T1 line	RSV B T2 line
*RSV A* samples (n = 87)	P/Total	83/87	72/87	83/87	0/87
	Sensitivity (95% CI)	95.4% (88.8%–98.2%)	82.8% (73.5%–89.3%)	95.4% (88.8%–98.2%)	—
	Specificity (95% CI)	—	—	—	100% (95.8%–100%)
*RSV B* samples (n = 94)	P/Total	93/94	87/94	1/94	92/94
	Sensitivity (95% CI)	98.9% (94.2%–99.8%)	92.6% (85.3%–96.4%)	—	97.9% (92.6%–99.4%)
	Specificity (95% CI)	—	—	98.9% (94.2%–99.8%)	—
Negative samples (n = 100)	P/Total	0/100	4/100	0/100	0/100
	Sensitivity (95% CI)	—	—	—	—
	Specificity (95% CI)	100% (96.3%–100%)	96% (90.2%–98.4%)	100% (96.3%–100%)	100% (96.3%–100%)

P, positive; CI, confidence interval; —, not applicable. RDT detects RSV without subtype discrimination; sensitivity was evaluated separately for RSV A and RSV B positive specimen groups. 95% CI by Wilson score method.

To assess the effect of viral load on assay performance, the clinical specimens were stratified according to the Ct values obtained using the Allplex SARS-CoV-2/FluA/FluB/RSV molecular comparator assay (Table 5). Specimens previously classified as RSV-positive by the Allplex Respiratory Panel 1 but not detected by the molecular comparator assay were analyzed separately as the not-detected group. For RSV A, the RSV A/B rh-RT-LAMP-NALF detection rates were 96.9% (31/32), 100.0% (27/27), 100.0% (16/16), and 87.5% (7/8) in the Ct < 25, 25≤ Ct < 30, 30≤ Ct < 35, and Ct ≥ 35 groups, respectively. The corresponding detection rates of the STANDARD Q RSV Ag Test were 93.8% (30/32), 92.6% (25/27), 75.0% (12/16), and 25.0% (2/8), respectively. In the four specimens not detected by the molecular comparator assay, the detection rates were 50.0% (2/4) for the RSV A/B rh-RT-LAMP-NALF assay and 75.0% (3/4) for the STANDARD Q RSV Ag Test. For RSV B, the RSV A/B rh-RT-LAMP-NALF detection rates were 98.3% (58/59), 100.0% (26/26), 100.0% (7/7), and 100.0% (1/1) across the respective Ct groups. The corresponding detection rates of the STANDARD Q RSV Ag Test were 93.2% (55/59), 96.2% (25/26), 85.7% (6/7), and 100.0% (1/1), respectively. The single specimen not detected by the molecular comparator assay was negative by both assays. Overall, the RSV A/B rh-RT-LAMP-NALF assay maintained high detection rates across the evaluated Ct ranges, whereas the STANDARD Q RSV Ag Test showed reduced detection at higher Ct values, particularly for RSV A.

**TABLE 5:** Detection performance of the RSV A/B rh-RT-LAMP-NALF assay and the STANDARD Q RSV Ag test according to Allplex™ SARS-CoV-2/FluA/FluB/RSV assay Ct-value ranges in RSV A- and RSV B-positive clinical specimens.

Clinical samples	Allplex™ SARS- CoV-2/FluA/FluB/RSV assay		RSV A/B rh-RT-LAMP-NALF assay		STANDARD Q RSV Ag test	
	Ct range	n	positive, n	detection rate	positive, n	detection rate
RSV A samples (n = 87)	Ct < 25	32	31	96.9%	30	93.8%
	25 ≤ Ct < 30	27	27	100.0%	25	92.6%
	30 ≤ Ct < 35	16	16	100.0%	12	75.0%
	Ct ≥ 35	8	7	87.5%	2	25.0%
	Not detected	4	2	50.0%	3	75.0%
RSV B samples (n = 94)	Ct < 25	59	58	98.3%	55	93.2%
	25 ≤ Ct < 30	26	26	100.0%	25	96.2%
	30 ≤ Ct < 35	7	7	100.0%	6	85.7%
	Ct ≥ 35	1	1	100.0%	1	100.0%
	Not detected	1	0	0.0%	0	0.0%

### 3.4 Cross-reactivity

Cross-reactivity was evaluated using 42 RSV-negative clinical specimens positive for other respiratory pathogens. The panel included three independent clinical specimens for each of 14 pathogens: Influenza A virus (FluA), Influenza B virus (FluB), parainfluenza virus 1 (PIV1), parainfluenza virus 2 (PIV2), parainfluenza virus 3 (PIV3), parainfluenza virus 4 (PIV4), human metapneumovirus (hMPV), human adenovirus (HAdV), human enterovirus (HEV), coronavirus OC43 (CoV-OC43), human bocavirus (HBoV), coronavirus 229E (CoV-229E), coronavirus NL63 (CoV-NL63), and human rhinovirus (HRV). All specimens were confirmed positive using the Allplex™ Respiratory Panel 1, 2, or 3 assays (Seegene, Seoul, Republic of Korea). The RSV A/B rh-RT-LAMP-NALF assay produced no T1 (RSV A) or T2 (RSV B) signal in any of the 42 specimens tested, indicating no detectable cross-reactivity with the respiratory pathogens evaluated (Table 6).

**TABLE 6:** Cross-reactivity of the RSV A/B rh-RT-LAMP-NALF assay against common respiratory pathogens.

Pathogen	n	Allplex assay	RSV A/B rh-RT-LAMP-NALF assay	
		Ct	T1 (RSV A)	T2 (RSV B)
Influenza A virus (FluA)	3	26.55/25.62/22.51	−/−/− (0/3)	−/−/− (0/3)
Influenza B virus (FluB)	3	25.10/38.40/27.75	−/−/− (0/3)	−/−/− (0/3)
Parainfluenza virus 1 (PIV1)	3	24.02/27.06/26.23	−/−/− (0/3)	−/−/− (0/3)
Parainfluenza virus 2 (PIV2)	3	30.03/26.96/29.18	−/−/− (0/3)	−/−/− (0/3)
Parainfluenza virus 3 (PIV3)	3	19.59/26.90/21.46	−/−/− (0/3)	−/−/− (0/3)
Parainfluenza virus 4 (PIV4)	3	27.38/26.24/30.25	−/−/− (0/3)	−/−/− (0/3)
Human metapneumovirus (hMPV)	3	23.70/28.08/26.37	−/−/− (0/3)	−/−/− (0/3)
Human adenovirus (HAdV)	3	17.45/26.58/24.67	−/−/− (0/3)	−/−/− (0/3)
Human enterovirus (HEV)	3	26.18/29.47/29.48	−/−/− (0/3)	−/−/− (0/3)
Coronavirus OC43 (CoV-OC43)	3	25.65/30.42/30.02	−/−/− (0/3)	−/−/− (0/3)
Human bocavirus (HBoV)	3	23.00/29.23/30.76	−/−/− (0/3)	−/−/− (0/3)
Coronavirus 229E (CoV-229E)	3	24.09/30.30/18.94	−/−/− (0/3)	−/−/− (0/3)
Coronavirus NL63 (CoV-NL63)	3	22.41/19.96/24.16	−/−/− (0/3)	−/−/− (0/3)
Human rhinovirus (HRV)	3	23.52/22.17/21.99	−/−/− (0/3)	−/−/− (0/3)

T1, RSV A detection line; T2, RSV B detection line; −, no signal detected.

## 4 Discussion

RSV subtype-specific detection is becoming increasingly important for molecular epidemiological surveillance and evaluation of preventive interventions. Because RSV A and RSV B may exhibit different circulation patterns and antigenic characteristics, subtype discrimination may provide clinically and epidemiologically relevant information (Nuttens et al., 2024; Staadegaard et al., 2021). In the present study, we developed and clinically validated a duplex rh-RT-LAMP-NALF assay for simultaneous subtype-specific detection of RSV A and RSV B using RNase HII-dependent blocked inner primers and multiplex lateral flow readout.

LAMP has emerged as a powerful molecular diagnostic platform because of its rapid amplification kinetics, isothermal operation, and minimal instrumentation requirements (Notomi et al., 2000). Previous RSV RT-LAMP studies demonstrated that LAMP-based assays can achieve clinically useful sensitivity within short turnaround times and may outperform antigen-based assays in low viral load specimens (Mahony et al., 2013; Ushio et al., 2005). More recently, multiplex RT-LAMP approaches for respiratory virus detection have been explored to enable syndromic point-of-care diagnostics (Lee et al., 2022). However, multiplex LAMP remains technically challenging because simultaneous amplification using multiple high-concentration primer sets substantially increases the risk of primer–primer interaction, spurious amplification, and false-positive signal generation (Yang et al., 2024). Several recent reviews have emphasized that non-specific amplification remains one of the principal limitations restricting the broader implementation of multiplex LAMP diagnostics, particularly in visually interpreted systems such as lateral flow assays (Jang and Kim, 2022; Kim et al., 2023).

To address this limitation, we incorporated RNase HII-dependent blocked inner primers into the duplex RT-LAMP system. The rhPCR strategy, originally described by Dobosy et al. (2011), employs primers containing both a single ribonucleotide residue and a 3′ blocking group, thereby preventing polymerase extension unless target-specific hybridization permits RNase HII-mediated cleavage. In the present study, this principle was applied to the inner primer design of the RSV A/B RT-LAMP-NALF assay. Specifically, the FIP of the RSV A primer set and the BIP of the RSV B primer set were designed as RNase HII-dependent blocked primers. Each blocked inner primer contained a single ribonucleotide residue within the target-complementary region and a 3′-BHQ1 blocking group at the terminal end. Accordingly, polymerase extension was permitted only after accurate target hybridization enabled RNase HII-mediated cleavage and release of the 3′ blocking group, whereas inefficient cleavage following non-target annealing or primer–primer interactions was expected to suppress non-specific extension. Supplementary Table S2 demonstrated that rhPCR-based blocked primers reduced non-specific lateral flow reactivity, with non-specific reactivity observed in 8 of 42 specimens under conventional RT-LAMP conditions but absent under rh-RT-LAMP conditions. These findings suggest that target-dependent primer activation can effectively suppress spurious amplification events generated during multiplex LAMP reactions.

The RSV A/B rh-RT-LAMP-NALF assay demonstrated analytical sensitivity down to 4 × 10^1^ copies/μL for both RSV A and RSV B plasmid standards, with no detectable cross-subtype signal. In serially diluted clinical specimens, the detection limit of the rh-RT-LAMP-NALF assay was approximately one dilution step less sensitive than that of the Allplex™ SARS-CoV-2/FluA/FluB/RSV assay, indicating slightly lower analytical sensitivity. Despite this, the assay achieved clinical sensitivity comparable to the Allplex™ RT-PCR assay for both RSV A (95.4% vs. 95.4%) and RSV B (97.9% vs. 98.9%), while demonstrating substantially higher sensitivity than the STANDARD Q RSV Ag Test (82.8% for RSV A; 92.6% for RSV B). In the RSV-negative panel, specificity was 100% on both the T1 and T2 lines, exceeding that of the STANDARD Q RSV Ag Test (96%). Cross-subtype specificity in the RSV-positive groups was 98.9% on the T1 line and 100% on the T1 line, respectively. These findings suggest that isothermal LAMP-based amplification can achieve diagnostic performance comparable to laboratory-based RT-PCR, while offering a clear advantage over antigen-based rapid diagnostic tests, particularly in specimens with low-to-moderate viral loads (Mahony et al., 2013). The substantially lower sensitivity of the STANDARD Q RSV Ag Test is consistent with the systematic review and meta-analysis reported by Chartrand et al. (2015), which demonstrated reduced sensitivity of RSV antigen detection assays in low viral load specimens and adult populations.

This study has several limitations. First, this was a retrospective study using archived specimens from a single institution. Because the clinical specimens were retrospectively and nonconsecutively selected to constitute predefined RSV A-positive, RSV B-positive, and RSV-negative groups, the possibility of selection and spectrum bias cannot be excluded. Prospective multicenter validation using consecutively enrolled patients in the intended clinical setting is therefore warranted to confirm assay performance across broader clinical settings. Second, the analytical LOD was determined using plasmid DNA standards rather than RNA standards and therefore reflects only the amplification and downstream lateral-flow detection steps. This DNA-based estimate does not account for losses during RNA extraction or inefficiencies and variability introduced during reverse transcription, all of which may reduce the sensitivity of the complete diagnostic workflow. Further evaluation using quantified RNA standards is required to determine an RNA-based analytical LOD, while whole-process testing of RNA-containing specimens from extraction through reverse transcription and LFA detection is needed to establish the LOD of the complete diagnostic workflow. Third, the RSV A/B rh-RT-LAMP-NALF assay maintained high detection rates even in specimens with Ct values ≥30, whereas the STANDARD Q RSV Ag Test showed reduced detection at higher Ct values. However, specimens with Ct values ≥30 accounted for only 27.6% (24/87) of the RSV A group and 8.5% (8/94) of the RSV B group. Therefore, the number of low-viral-load specimens was insufficient to reliably evaluate assay performance near the detection limit, and further validation using a larger number of high-Ct clinical specimens is required. Finally, extraction-free sample processing was not evaluated, and further studies will be needed to simplify sample preparation for point-of-care applications. Third, the applicability of the rhPCR-based blocked primer strategy to higher-order multiplex respiratory panels remains to be determined.

Overall, the present study demonstrates that RNase HII-dependent blocked primer activation can effectively suppress non-specific amplification in duplex RT-LAMP-NALF, enabling simultaneous subtype-specific detection of RSV A and RSV B with diagnostic performance comparable to laboratory-based RT-PCR and substantially superior to antigen-based rapid tests. This platform provides rapid, minimal-instrumentation, and subtype-resolved RSV diagnosis suitable for decentralized settings, and the underlying rhPCR-based specificity strategy may be further extended to broader multiplex respiratory pathogen panels.

## Data Availability

The raw data supporting the conclusions of this article are included in the Supplementary Material.
