# Supplementary material for: Rapid subtype-specific detection of respiratory syncytial virus A and B using an RNase HII-dependent RT-LAMP lateral flow assay
Source: Front Chem. 2026 Sep 10;14:1942908. doi: 10.3389/fchem.2026.1942908 (PMC13601926; doi:10.3389/fchem.2026.1942908)
Supplement: Supplementary file 1 [file Table1.docx]

**TABLE S1.** In silico analysis of potential secondary structures and primer-dimer formation within the complete duplex RSV A/B rh-RT-LAMP-NALF primer set.

| **Primer** | **Length (nt)** | **Hairpin** | | **Homodimer** | | **Strongest predicted heterodimer** | | |
| --- | --- | --- | --- | --- | --- | --- | --- | --- |
|  |  | **ΔG at 62°C (kcal/mol)** | **Tm (°C)** | **ΔG at 62°C (kcal/mol)** | **Tm (°C)** | **Primer pair** | **ΔG at 62°C (kcal/mol)** | **Tm (°C)** |
| RSV A F3 | 23 | — | — | -1.37 | -8.3 | A F3–B LF | -1.40 | -25.5 |
| RSV A B3 | 19 | — | — | 0.34 | 17.2 | A B3–A BIP | -5.14 | 36.0 |
| RSV A rhFIP | 44 | 0.49 | 58.3 | 5.53 | 30.2 | A rhFIP–B rhBIP | -2.70 | 25.4 |
| RSV A BIP | 44 | — | — | -0.79 | 15.2 | A BIP–A B3 | -5.14 | 36.0 |
| RSV A LF | 22 | 0.06 | 60.5 | -3.21 | 33.0 | A LF–A LB | -2.38 | 1.6 |
| RSV A LB | 25 | — | — | -1.85 | 9.6 | A LB–A BIP | -2.38 | 1.6 |
| RSV B F3 | 18 | — | — | -0.18 | -5.2 | B F3–A BIP | -2.49 | 25.7 |
| RSV B B3 | 19 | — | — | -0.62 | -18.2 | B B3–A BIP | -2.07 | -3.0 |
| RSV B FIP | 50 | — | — | 4.02 | 30.3 | B FIP–B LF | -2.70 | 21.9 |
| RSV B rhBIP | 49 | 0.30 | 58.7 | 0.47 | 13.7 | B rhBIP–A rhFIP | -2.70 | 25.4 |
| RSV B LF | 25 | — | — | -2.45 | -6.3 | B LF–B FIP | -2.70 | 21.9 |
| RSV B LB | 25 | — | — | -1.62 | -8.0 | B LB–A B3 | -2.19 | 7.4 |

Analysis conditions. Primer3-py v2.3.0; monovalent cations, 50 mM; divalent cations, 8 mM; dNTPs, 1.4 mM; oligonucleotide concentration, 1.6 µM (conservative upper concentration used for all calculations); calculation temperature, 62°C.

Sequence treatment. For RSV A rhFIP and RSV B rhBIP, the ribonucleotide was entered as the corresponding DNA base (A), and the appended CTTA or TTTG sequence was included. BHQ1, FAM, DIG, and biotin modifications were excluded because these non-nucleotide labels cannot be modeled by Primer3-py.

Interpretation. No predicted self-dimer or heterodimer had a melting temperature approaching the 62°C reaction temperature. Hairpins predicted for RSV A rhFIP, RSV A LF, and RSV B rhBIP had Tm values of 58.3–60.5°C but positive ΔG values at 62°C, indicating that they were not thermodynamically stable at the reaction temperature.

**TABLE S2.** Reduction of non-specific lateral flow signals by RNase HII-dependent blocked primers in RSV A/B RT-LAMP-NALF.

| **Pathogen** | **RSV A/B rh-RT-LAMP-NALF assay** | | **RSV A/B RT-LAMP-NALF assay** | |
| --- | --- | --- | --- | --- |
|  | **rh-RT-LAMP T1** | **rh-RT-LAMP T2** | **RT-LAMP T1** | **RT-LAMP T2** |
| Influenza A virus (FluA) | −/−/− (0/3) | −/−/− (0/3) | −/−/− (0/3) | −/−/− (0/3) |
| Influenza B virus (FluB) | −/−/− (0/3) | −/−/− (0/3) | −/−/− (0/3) | −/−/− (0/3) |
| Parainfluenza virus 1 (PIV1) | −/−/− (0/3) | −/−/− (0/3) | −/−/− (0/3) | −/−/− (0/3) |
| Parainfluenza virus 2 (PIV2) | −/−/− (0/3) | −/−/− (0/3) | **+/+/− (2/3)** | −/−/− (0/3) |
| Parainfluenza virus 3 (PIV3) | −/−/− (0/3) | −/−/− (0/3) | −/−/− (0/3) | −/−/− (0/3) |
| Parainfluenza virus 4 (PIV4) | −/−/− (0/3) | −/−/− (0/3) | −/−/− (0/3) | **−/−/+ (1/3)** |
| Human metapneumovirus (hMPV) | −/−/− (0/3) | −/−/− (0/3) | −/−/− (0/3) | **−/−/+ (1/3)** |
| Human adenovirus (HAdV) | −/−/− (0/3) | −/−/− (0/3) | −/−/− (0/3) | −/−/− (0/3) |
| Human enterovirus (HEV) | −/−/− (0/3) | −/−/− (0/3) | **−/−/+ (1/3)** | **−/−/+ (1/3)** |
| Coronavirus OC43 (CoV-OC43) | −/−/− (0/3) | −/−/− (0/3) | −/−/− (0/3) | **+/ −/− (1/3)** |
| Human bocavirus (HBoV) | −/−/− (0/3) | −/−/− (0/3) | −/−/− (0/3) | −/−/− (0/3) |
| Coronavirus 229E (CoV-229E) | −/−/− (0/3) | −/−/− (0/3) | −/−/− (0/3) | **−/−/+ (1/3)** |
| Coronavirus NL63 (CoV-NL63) | −/−/− (0/3) | −/−/− (0/3) | **−/−/+ (1/3)** | −/−/− (0/3) |
| Human rhinovirus (HRV) | −/−/− (0/3) | −/−/− (0/3) | −/−/− (0/3) | **−/+/− (1/3)** |

† Non-specific signals (bold) were observed in 8 of 42 specimens under conventional RT-LAMP, compared with 0 of 42 under rh-RT-LAMP. One HEV-positive specimen produced non-specific signals on both T1 and T2 and was counted once. Three clinical specimens per pathogen were tested. T1, RSV A line; T2, RSV B line; −, negative; +, positive.
